# A Case of Iatrogenic Benign Cyst Infection Arising at the Anastomosis after Functional End-to-End Anastomosis for Cecal Cancer

**DOI:** 10.70352/scrj.cr.25-0770

**Published:** 2026-04-04

**Authors:** Hiromitsu Imataki, Masaoki Hattori, Marie Hotta, Akihiro Hirata, Akihiro Tomida, Takuya Arakawa, Jumpei Shibata, Marika Suzuki, Hideharu Shintomi, Motoi Yoshihara, Yoshikazu Mizoguchi

**Affiliations:** 1 Department of Surgery, Nishichita General Hospital, Tokai, Aichi, Japan; 2 Department of Pathology, Nishichita General Hospital, Tokai, Aichi, Japan

**Keywords:** anastomosis, cyst, implantation cyst, duplication cyst, functional end-to-end anastomosis, double-stapling technique, laparoscopy

## Abstract

**INTRODUCTION:**

Implantation cysts are iatrogenic cystic lesions that develop at anastomotic sites, most commonly following double-stapling technique anastomoses.

**CASE PRESENTATION:**

A 77-year-old woman underwent laparoscopic ileocecal resection with functional end-to-end anastomosis for cecal cancer. Contrast-enhanced CT performed 6 months postoperatively revealed a 25-mm cystic lesion contiguous with the anastomotic stapler line, protruding extramurally from the bowel wall. The lesion was diagnosed as an iatrogenic benign cyst at the anastomotic site, and careful observation was adopted. Six years and six months after surgery, the cyst became infected. Elective laparoscopic local resection was performed, resulting in complete resolution.

**CONCLUSIONS:**

Histopathological examination demonstrated features resembling an enteric duplication cyst, suggesting that an isolated intestinal structure became cystic due to mucin production. To our knowledge, no cases reported since 1990 have described cyst formation with subsequent infection following functional end-to-end anastomosis. This case does not meet the classical definition of an implantation cyst. With the increasing use of linear staplers for intracorporeal anastomoses, similar lesions may be encountered. Accumulation of such cases may support recognition of this entity as an “anastomotic duplication cyst”.

## Abbreviations

ADC
: anastomotic duplication cyst
DST
: double-stapling technique
FEEA
: functional end-to-end anastomosis
IC
: implantation cyst

## INTRODUCTION

ICs are cystic lesions that develop at gastrointestinal anastomotic sites. First described by Dukes, ICs are lesions in which the gastrointestinal epithelial mucosa is displaced and implanted into the submucosal layer during anastomotic manipulation, subsequently producing mucus and forming a cyst.^1)^ With the increasing use of the DST for intracorporeal anastomosis, IC reports have been accumulating.^2)^

We encountered a cystic lesion that developed iatrogenically at the anastomosis site following FEEA for cecal cancer that was initially managed with observation.

To date, no cases of cystic lesions arising at an anastomosis created by the FEEA have been reported, nor have such lesions become infected or perforated.

## CASE PRESENTATION

A 77-year-old woman with a history of cecal cancer and comorbid diabetes mellitus, hypertension, and hyperlipidemia had undergone laparoscopic ileocecal resection with D3 lymph node dissection and FEEA 6 years and 6 months earlier. After exteriorization of the anastomotic segment, an FEEA was constructed between the remaining ileum and the ascending colon using a ReliaMax linear stapler cartridge (Covidien Japan, Tokyo, Japan; GIA80-3.8, GIA8038RL; blue cartridge with 2 staple rows). The operative time was 2 h and 2 min, and intraoperative blood loss was 59 mL.

Postoperatively, the patient did not develop a fever exceeding 38°C and had no abdominal symptoms suggestive of anastomotic leakage. Although the C-reactive protein level was elevated to 19.7 mg/dL on POD3, the white blood cell count remained within the normal range (4650/μL), and no additional antibiotics were required beyond prophylactic administration within the first 48 h after surgery. The postoperative hospital stay was prolonged due to an umbilical wound infection; however, this resolved following incision and drainage, and the patient was discharged home on POD13.

**Fig. 1:**
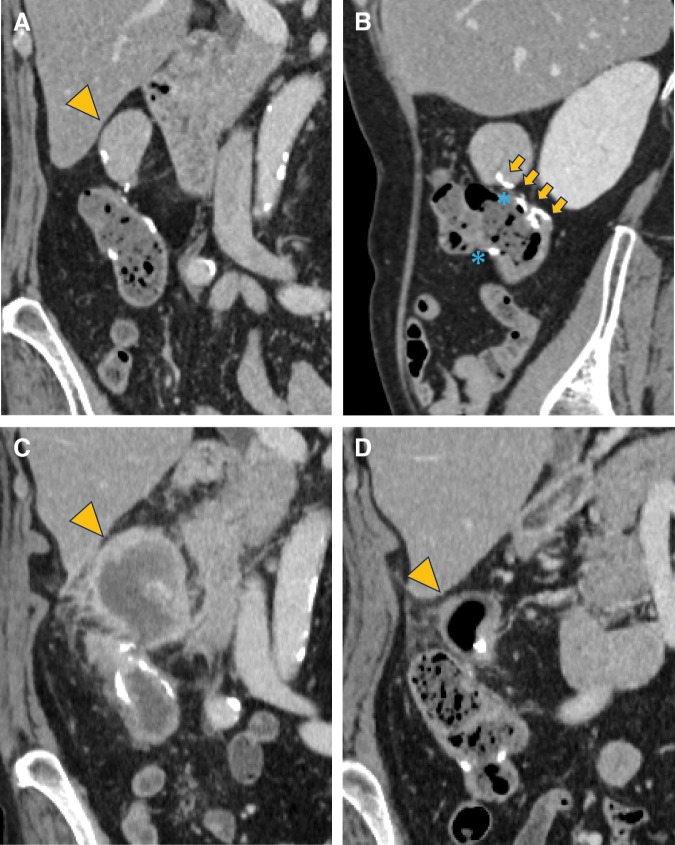
Contrast-enhanced CT findings. (**A**) Five years after surgery, before inflammation onset: A 35-mm dumbbell-shaped low attenuation mass protruding extramurally from the FEEA stapler line (arrowhead). The mass had a smooth surface, homogeneous internal contents, and no contrast enhancement (coronal view). (**B**) The cyst appears to be contiguous with the stapler line of the stump. The stapler line of the common orifice (asterisk) and that of the stump (arrows) are indicated (sagittal views). (**C**) At the time of inflammatory onset, 6 years and 6 months postoperatively: The mass (arrowhead) has enlarged to 44 mm, with wall thickening and increased surrounding fat stranding. No solid internal components, wall disruption, or perilesional fluid collection suggestive of rupture is observed. (**D**) After conservative treatment: The mass (arrowhead) has decreased to 35 mm, with wall thickening and adjacent fat stranding inflammation. Air is newly visible within the cyst. FEEA, functional end-to-end anastomosis

**Fig. 2:**
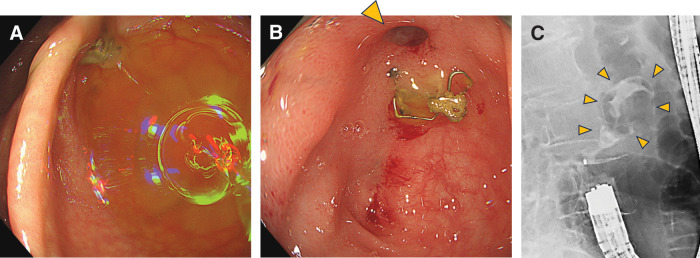
Colonoscopic findings. (**A**) Three years after surgery: Staple material is visible at the anastomosis. No fistula or other abnormalities are detected. (**B**) After conservative treatment: A 2-mm fistulous opening is seen adjacent to the anastomotic staple line (arrowhead). (**C**) After conservative treatment: Fistulography demonstrates contrast medium entering the cyst cavity (arrowheads).

Histopathological examination revealed well-differentiated tubular adenocarcinoma (pT3, Ly1, v0, N0, M0; pStage IIA). No adjuvant chemotherapy was administered, and postoperative surveillance consisted of regular blood tests and imaging studies. Contrast-enhanced CT performed 6 months after surgery revealed a 25-mm dumbbell-shaped cystic lesion contiguous with the anastomotic stapler line and protruding extramurally from the bowel wall. Because the lesion showed no solid components or invasive features, it was diagnosed as an iatrogenic benign anastomotic cyst, and observation was continued. Over the subsequent 5 years, the cyst gradually enlarged to 35 mm without associated symptoms (**Fig. 1A**) or abnormal findings on lower gastrointestinal endoscopy (**Fig. 2A**), making anastomotic recurrence unlikely. On sagittal CT images, the cyst appeared contiguous with the stapler line of the stump (**Fig. 1B**).

At 6 years and 6 months after surgery, the patient presented with a 1-week history of intermittent right lower abdominal pain and fever. On admission, her body temperature was 38.3°C, and physical examination revealed localized tenderness in the right lower quadrant without peritoneal signs. Laboratory tests demonstrated elevated inflammatory markers, with a C-reactive protein level of 16.5 mg/dL, while tumor marker levels were within the normal range. CT revealed enlargement of the cyst to 44 mm, accompanied by wall thickening and surrounding fat stranding, findings consistent with cyst infection (**Fig. 1C**).

**Fig. 3:**
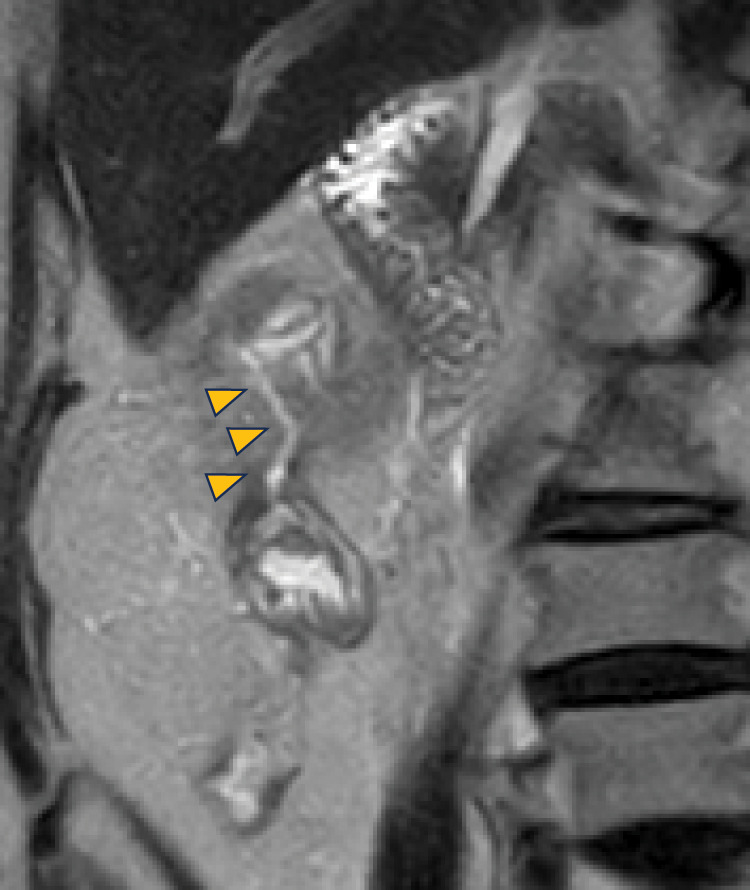
MRI findings (coronal, T2-weighted imaging). MRI performed following conservative treatment demonstrates a 35-mm mass protruding extramurally and in continuity with the anastomotic site. The cyst lumen demonstrated high T2 signal intensity without solid components. Fistulous communication between the cyst cavity and anastomotic bowel lumen is observed (arrowheads).

Conservative treatment consisting of fasting and antibiotic therapy was initiated. The fever resolved and abdominal pain improved by day 2 of treatment, and both pain and tenderness had completely disappeared by day 4. The C-reactive protein level rapidly decreased to 2.2 mg/dL by day 6. Follow-up CT after conservative treatment showed a reduction in cyst size to 35 mm, with newly observed intralesional air (**Fig. 1D**). Subsequent MRI (**Fig. 3**) and colonoscopy (**Fig. 2B** and **2C**) confirmed fistulous communication between the cyst cavity and the anastomotic lumen.

Based on these findings, the lesion was preoperatively diagnosed as an infected benign cyst iatrogenically generated at the FEEA anastomosis that had ruptured into the anastomotic lumen. Given the risk of recurrent infection and the limited drainage through the small fistula, surgical resection was planned.

**Fig. 4:**
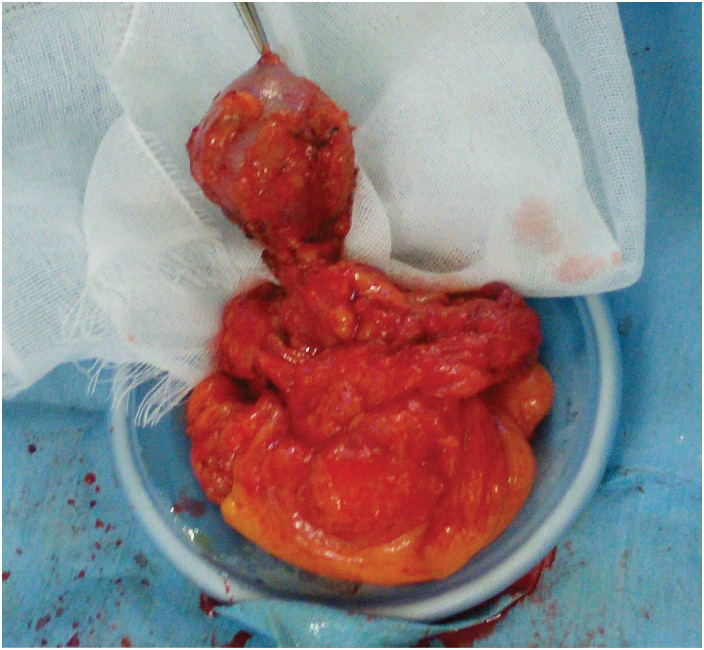
Intraoperative findings. The cyst was palpable as a soft, elastic mass. During dissection, the lesion was easily separated from the surrounding tissues and pedunculated, with only the fistulous tract remaining attached. The cyst had a smooth serosal surface that was preserved without evidence of invasion into adjacent structures.

Laparoscopic exploration revealed dense adhesions surrounding the anastomotic site. After mobilization, the anastomosis was exteriorized through a small umbilical incision, revealing a smooth, soft, pedunculated cyst arising from the anastomosis (**Fig. 4**). The cyst was excised together with a small portion of the anastomotic bowel wall, including the fistulous tract, and the defect was closed using interrupted sutures. The operative time was 3 h and 26 min, and blood loss was 10 mL.

**Fig. 5:**
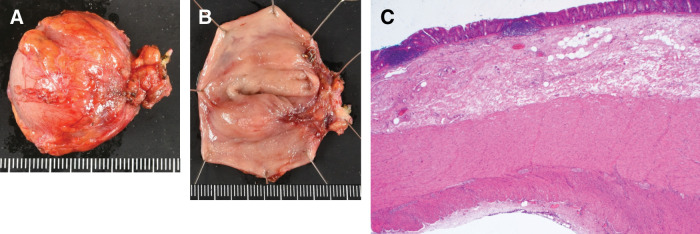
Histopathological findings. (**A**) Gross appearance, serosal surface: The cyst surface resembles serosa, similar to normal bowel. (**B**) Gross appearance, mucosal surface: The cyst lumen appears lined by mucosal epithelium, resembling normal bowel mucosa. (**C**) Histologic section: The cyst wall exhibits a normal, layered intestinal structure—mucosa, submucosa, muscularis propria, subserosa, and serosa—identical to that of the native bowel wall.

Histopathological examination demonstrated that the cyst wall was composed of all layers of the intestinal wall, without evidence of atypia or malignancy (**Fig. 5**). The postoperative course was uneventful, and the patient was discharged on POD9. No recurrence was observed during 1 year of follow-up.

## DISCUSSION

A literature search of Ichushi-Web (Japan Medical Abstracts Society) using the Japanese terms “cyst,” “anastomosis,” and “functional end-to-end anastomosis,” and of PubMed using “cyst,” “anastomosis,” “anastomotic,” “functional end-to-end anastomosis,” and “FEEA” identified 35 anastomotic cyst cases reported since 1990, including 18 and 17 in English^3–8)^ and Japanese, respectively. All previously reported lesions were ICs, and no reports described cystic lesions arising at FEEA anastomoses (**Table 1**). IC reports following prolapse and hemorrhoid procedures were excluded because they did not involve gastrointestinal anastomosis.

**Table 1:** Reported cases of anastomotic cysts (1990–2025)

Year	Author	Age	Sex	Primary disease	Anastomosis	Symptom	Major axis of cysts (mm)	Diagnosis	Treatment	Location of cysts	Pathological findings
2008	Honda^3)^	70	M	Rectal cancer	DST	—	25–35	IC	F/U	Submucosa	NA
		69	M	Rectal cancer	DST	—	25–35	IC	F/U	Submucosa	NA
		70	M	Rectal cancer	DST	—	25–35	IC	F/U	Submucosa	NA
		70	F	Rectal cancer	DST	—	25–35	IC	F/U	Submucosa	NA
2010	Katsumata^4)^	55	M	Rectal cancer	DST	ND	ND	Local recurrence in IC	Laparotomic LAR	Submucosa	Colonic mucosa and local recurrence
		68	F	Rectal cancer	DST	ND	ND	IC	Trans anal dorenage^*1^	ND	NA
		63	F	Rectal cancer	DST	—	ND	IC	F/U	ND	NA
		62	M	Rectal cancer	DST	—	ND	IC	F/U	ND	NA
		64	F	Rectal cancer	DST	—	ND	IC	F/U	ND	NA
		75	M	Rectal cancer	DST	—	ND	IC	F/U	ND	NA
		76	M	Rectal cancer	DST	—	ND	IC	F/U	ND	NA
		66	M	Rectal cancer	Hartmann	—	ND	IC	F/U	ND	NA
		50	M	Rectal cancer	DST	—	ND	IC	F/U	ND	NA
2023	Fujita^5)^	47	M	Rectal cancer	DST	—	ND	Adenocarcinoma arising from an IC	Laparoscopic TPE	Submucosa	Adenocarcinoma in cyst
2024	Iwasa^6)^	70	M	Acute gastric ulcer	Gastrojejunal^*2^	—	20	IC	F/U	Submucosa	NA
2024	Shimada^7)^	70	F	Rectal cancer	DST^*3^	—	60	Adenocarcinoma arising from an IC	Laparotomic APR	Submucosa	Adenocarcinoma in cyst
2024	Kameoka^8)^	81	F	Rectal cancer	DST	—	30	IC	Endoscopic fenestration	Outside the muscle layer in contact with the rectal wall	NA
2025	Imataki	77	F	Cecal cancer	FEEA	Fever and abdominal pain	44	ADC	Laparoscopic local resection	Outside the anastomotic site	Wall structure similar that of normal intestines

^*1^ Cyst recurrent after treatment.

^*2^ The images suggest a hand-sewn side-to-side anastomosis.

^*3^ The images suggest a DST anastomosis.

ADC, anastomotic duplication cyst; APR, abdominoperineal resection; DST, double-stapling technique; F, female, FEEA, functional end-to-end anastomosis; F/U, follow-up; IC, implantation cyst; LAR, low anterior resection; M, male; NA, not applicable; ND, not described; TPE, total pelvic exenteration

**Fig. 6:**
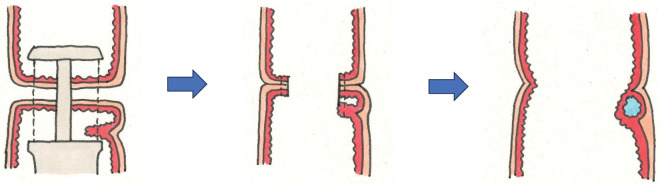
Mechanism of implantation cyst formation in DST anastomosis. ICs arise when the circular stapler used in the DST inadvertently incorporates excessive rectal mucosa. DST, double-stapling technique; ICs, implantation cysts

Recent ICs have been reported almost exclusively at DST anastomoses, with only single cases at Hartmann stump^4)^ and at a gastrojejunostomy site.^6)^ Katsumata et al. retrospectively reviewed 448 low anterior resections and identified ICs in 9 patients (2%), concluding that device characteristics and surgical technique influence cyst formation.^4)^ ICs arise when a circular stapler used during DST inadvertently incorporates excess rectal mucosa (**Fig. 6**).^9)^ Because the circular stapler approximates the luminal diameter and is introduced transanally, the mucosal entrapment risk is higher, and fine manual adjustment is often not feasible during intracorporeal DST anastomosis.

Conversely, FEEA is performed exclusively with linear staplers in 2 stages: side-to-side anastomosis using mucosa-side stapling and common entry hole closure using serosa-side stapling. IC formation theoretically occurs when excess mucosa is incorporated during mucosa-side stapling.^9)^ Nevertheless, because linear stapler anvils are relatively narrow compared with the bowel lumen diameter and FEEA is often performed extracorporeally, manual adjustment is easier, which reduces the likelihood of mucosal entrapment compared with DST.

ICs are cysts formed when intestinal mucosal epithelium is misplaced into the submucosa during the anastomotic procedure, where it continues to secrete mucus and thereby forms a cyst.^1)^ Almost all previously reported ICs were submucosal cysts, except for a single atypical case reported by Kameoka et al.,^8)^ in which an IC at a rectal DST anastomosis lay external to the muscularis propria and abutted the rectal wall. Although a few atypical cases exist, ICs are fundamentally submucosal.

This case was atypical because the cyst protruded extramurally in continuity with the FEEA stapler line. Histopathologically, the lesion exhibited a complete intestinal wall structure extending outward from the fistulous area. Rather than mucosal misplacement as in ICs, the lesion appeared to represent a closed intestinal segment that had undergone mucinous dilatation and resembled an enteric duplication cyst. Ladd et al. defined enteric duplication cysts as gastrointestinal malformations lined by gastrointestinal epithelium, surrounded by smooth muscle, and adjacent to the gastrointestinal tract.^10)^ Because no similar cases have been reported, classification was difficult, and we ultimately diagnosed the lesion as a benign cyst resembling a duplication cyst iatrogenically created at the FEEA anastomosis.

**Fig. 7:**
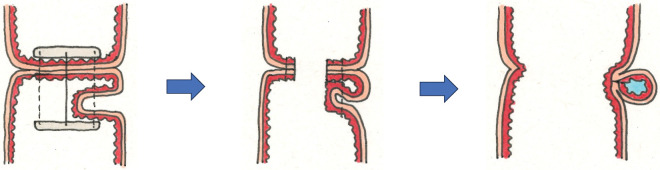
Proposed mechanism of duplication-like anastomotic cyst formation in FEEA (mucosa-side stapling pattern). If the full-thickness bowel wall is inadvertently folded and incorporated during mucosa-side stapling, a closed cavity that retains the full intestinal wall architecture may form, resulting in a duplication-like cystic structure. FEEA, functional end-to-end anastomosis

**Fig. 8:**
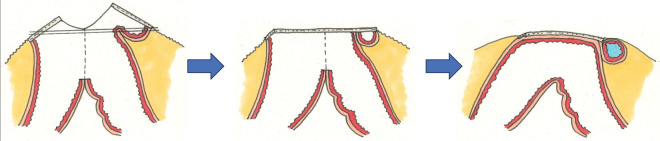
Proposed mechanism of duplication-like anastomotic cyst formation in FEEA (serosa-side stapling pattern). If the full-thickness bowel wall is inadvertently folded and incorporated during the serosa-side closure of a common enterotomy, a closed space with an intact full-layer bowel structure may similarly arise, producing a duplication-like cystic lesion. FEEA, functional end-to-end anastomosis

Kameoka et al. proposed that ICs located external to the muscularis propria result from full-thickness invagination during the DST.^8)^ If a similar mechanism occurred during FEEA, it may have resulted from mucosa-side linear stapling (**Fig. 7**). Alternatively, the lesion may have formed during serosa-side stapling of the common channel closure if full-thickness bowel layers were inadvertently incorporated (**Fig. 8**). Based on the imaging findings, the cyst appeared contiguous with the stump stapler line, suggesting that it originated from serosa-side stapling. In both scenarios, device mechanics and procedural techniques likely contributed to cyst formation, as observed in ICs.

In Japan, postoperative surveillance for gastrointestinal malignancies generally follows guideline recommendations. Of the 35 reported IC cases, 28 were asymptomatic and were discovered incidentally during surveillance imaging. Three cases of adenocarcinoma arising within ICs have been reported^4,5,7)^: 1 occurred 14 months after surgery and was considered a local recurrence,^4)^ whereas the other 2 occurred 26 and 30 years postoperatively,^5,7)^ respectively. These reports suggest the potential for malignant transformation and support long-term surveillance.

In the present case, an iatrogenic duplication cyst at the anastomotic site was detected on the initial postoperative CT performed 6 months after surgery, and infection developed 6 years later. Although antibiotic therapy was initially effective, surgery was performed because of the risk of recurrent infection and potential malignant transformation. In addition, the pedunculated, extramurally protruding cyst was suitable for curative minimally invasive laparoscopic local resection. If similar cases arise in the future, this report may provide valuable information for surgeons.

As DST-based intracorporeal anastomosis has become more common, the number of IC reports has correspondingly increased. With the increasing adoption of intracorporeal anastomosis using linear staplers, including the FEEA, overlapping anastomosis, and delta-shaped anastomosis, similar cyst formation may occur. Although generalization based on a single novel case is difficult, the accumulation of similar cases may justify the term ADC.

## CONCLUSIONS

In this case, the cystic lesion arising at the FEEA anastomosis did not meet the definition of an IC and exhibited features resembling an enteric duplication cyst. As intracorporeal anastomoses using linear staplers become more widespread, similar lesions may emerge. Although iatrogenic anastomotic cysts are generally benign and often observed, they rarely become infected or undergo malignant transformation. In this case, the morphology allowed successful laparoscopic-assisted local resection, which was curative and minimally invasive and therefore constituted an appropriate therapeutic choice.
